# Cepharanthine Hydrochloride Improves Cisplatin Chemotherapy and Enhances Immunity by Regulating Intestinal Microbes in Mice

**DOI:** 10.3389/fcimb.2019.00225

**Published:** 2019-06-26

**Authors:** Pengjun Zhou, Ziyao Li, Dandan Xu, Ying Wang, Qi Bai, Yue Feng, Guifeng Su, Pengxiao Chen, Yao Wang, Huizhong Liu, Xiaogang Wang, Rong Zhang, Yifei Wang

**Affiliations:** ^1^Guangdong Provincial Key Laboratory of Bioengineering Medicine, Guangzhou Jinan Biomedicine Research and Development Center, College of Life Science and Technology, Jinan University, Guangzhou, China; ^2^Guangdong Food and Drug Vocational College, Guangzhou, China; ^3^College of Food Science and Technology, Zhongkai University of Agriculture and Engineering, Guangzhou, China; ^4^Department of Hepatobiliary Surgery, Xijing Hospital, Fourth Military Medical University, Xi'an, China; ^5^Department of Mathematics and Statistics, York University, Toronto, ON, Canada; ^6^State Key Laboratory of Oncology in South China and Collaborative Innovation Center for Cancer Medicine, Sun Yat-sen University Cancer Center, Guangzhou, China

**Keywords:** cepharanthine hydrochloride, esophageal cancer, gut microbes, chemotherapy, intestinal mucosal immunity

## Abstract

Chemotherapy is one of the major treatment strategies for esophageal squamous cell carcinoma (ESCC). Unfortunately, most chemotherapeutic drugs have significant impacts on the intestinal microbes, resulting in side effects and reduced efficiency. Therefore, new strategies capable of overcoming these disadvantages of current chemotherapies are in urgent need. The natural product, Cepharanthine hydrochloride (CEH), is known for its anticancer and immunoregulatory properties. By sequencing the V4 region of 16S rDNA, we characterized the microbes of tumor-bearing mice treated with different chemotherapy strategies, including with CEH. We found that CEH improved the therapeutic effect of CDDP by manipulating the gut microbiota. Through metagenomic analyses of the microbes community, we identified a severe compositional and functional imbalance in the gut microbes community after CDDP treatment. However, CEH improved the effect of chemotherapy and ameliorated CDDP treatment-induced imbalance in the intestinal microbes. Mechanically, CEH activated TLR4 and MYD88 innate immune signaling, which is advantageous for the activation of the host's innate immunity to exert a balanced intestinal environment as well as to trigger a better chemotherapeutic response to esophageal cancer. In addition, TNFR death receptors were activated to induce apoptosis. In summary, our findings suggest that chemotherapy of CDDP combined with CEH increased the effect of chemotherapy and reduced the side effects on the microbes and intestinal mucosal immunity. We believe that these findings provide a theoretical basis for new clinical treatment strategies.

## Introduction

Esophageal squamous cell carcinoma (ESCC) is the main histological type of esophageal cancer, accounting for 90% of all esophageal cancers, and is one of the most common fatal cancers (McGuire, 2016). While the incidence of ESCC in men has declined globally, its incidence in women has increased (Wang et al., 2018). Because ESCC is mostly diagnosed in the advanced stages or during metastasis, patients cannot undergo radical surgery. Thus, the remaining therapeutic options for these patients are radiotherapy and chemotherapy. However, chemotherapy has a significant impact on the intestinal microbes and may lead to intestinal mucosal inflammation, which is usually caused by intestinal ecological disorders (Montassier et al., 2015; Ichim et al., 2018; Perales-Puchalt et al., 2018). Therefore, it is important to develop new treatment strategies or drugs for ESCC patients in order to improve the intestinal dysbiosis caused by chemotherapy.

Platinum derivatives are one of the most commonly used chemotherapy drugs. According to the guidelines for esophageal and esophagogastric cancer published by the National Comprehensive Cancer Network, cisplatin (CDDP) is used as a first-line therapy alone or in combination with other chemotherapeutic drugs in treatment of multiple tumors, including ovarian, liver, non-small cell lung, cervical, pancreatic, and prostate cancers. However, the non-specific effect of CDDP, which crosslinks DNA to interfere with DNA replication, induces multiple adverse effects. Furthermore, CDDP exerts antibiotic effects on both Gram-positive and Gram-negative bacterial strains (Joyce et al., 2010). The imbalance of distribution and function in the intestinal microbes caused by CDDP leads to severe intestinal mucosal damage and contributes to adverse events, including similar negative behavioral changes and concurrent gastrointestinal symptoms. These side effects of CDDP severely deteriorate the cancer patients' quality of life and even precludes the progress of treatment (Jordan et al., 2018).

Over the past decade, our understanding of the distribution and function of the gut microbes has been greatly enhanced, to the extent that it is now considered a new organ and thought to play an important role in the immune system. The interaction between commensal bacteria and host cells in the gut is tightly regulated to maintain homeostasis and distinguish commensal microorganisms from pathogens (Perez-Lopez et al., 2016). A series of pattern recognition receptors (PRRs) expressed by host intestinal mucosal immune cells and non-immune cells, including Toll-like receptors (TLRs) and NOD-like receptors (NLRs), can detect pathogen-associated molecular patterns (PAMPs). Among them, the identification of commensal bacteria based on TLRs represents the crucial aspects of symbiosis between host and microbiota (Weiss et al., 2004; Sivick et al., 2014). In addition to these innate responses, the activation of adaptive immunity leading to the production of protective sIgA is another immune mechanism that can protect the host from infection by enteric bacteria. Research on the relationship between intestinal microbes and host immunity is fairly scarce, especially in the case of chemotherapy-induced intestinal microbes' dysfunction. Efforts to elucidate the effects of these changes are critical for the design of new chemotherapy strategies. Although some progress has been made, due to the lack of tools and human volunteers, studies on clarifying the relationship between chemotherapy and intestinal microbes still face numerous challenges. Therefore, at present, mouse models are commonly used to mimic human intestinal microbes composition and functional changes.

Dibenzylisoquinoline alkaloid monomer derivative Cepharanthine and its semi-synthetic compound Cepharanthine hydrochloride (CEH) have been previously reported to have many biological effects, including anti-inflammatory and anti-tumor, and enhances leukopenia caused by chemotherapy (Furusawa and Wu, 2007; Zhou et al., 2012, 2017; Matsuda et al., 2014; Kao et al., 2015). Clinically, it is mainly used to prevent leukopenia caused by radiotherapy and chemotherapy in cancer patients (Ushiki et al., 1988; Ohta and Morita, 1990; Suzuki et al., 1990, 1992). As we reported in our previous work, CEH could effectively reverse the MDR-mediated CDDP resistance of ESCC cells *in vitro* and *in vivo* (Zhou et al., 2017). Other studies have reported that CEH regulates immunity and possesses anti-inflammatory effects (Kondo et al., 1992; Murakami et al., 2003; Nagano et al., 2003; Seubwai et al., 2010; Rogosnitzky and Danks, 2011; Uto et al., 2011; Kao et al., 2015; Paudel et al., 2016). Nevertheless, there is no research on the effects of CEH on intestinal mucosal immunity or regulation of intestinal microbes. Therefore, in this study, we established a mouse model of xenograft tumors, which mimicked the disturbance state of intestinal microbes treated by CDDP in humans. By using this animal model, we investigated the role of CEH in maintaining the balance of intestinal microbes and host intestinal mucosal immunity during chemotherapy. We then performed 16S rDNA V4 variable region sequencing and bioinformatics analyses, as well as metagenomic analysis using the IlluminaHiseqPE platform to identify microbes and microbes functions that are modulated following CDDP chemotherapy in the presence or absence of CEH. Our study suggests a new chemotherapy strategy for reducing the adverse effects of CDDP monotherapy through combination with CEH, which may be beneficial in regulating intestinal microbiota.

## Materials and Methods

### Cell Lines and Culture Conditions

Human ESCC cell line Eca109 cells were maintained in RPMI 1640 Basic Medium (Gibco™ 11875119) with 10% fetal bovine serum (ExCell FSP500) at 37°C in a humidified atmosphere of 5% CO_2_.

### Mice

**Figure 1:**
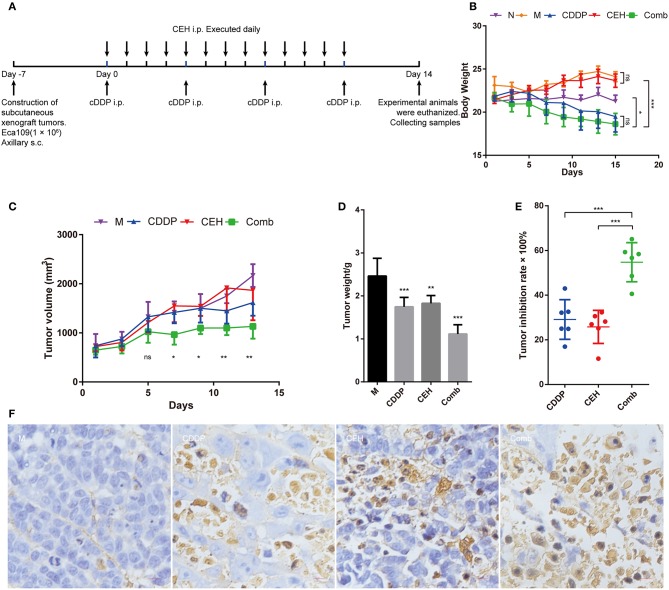
CEH combined with CDDP chemotherapy inhibits the growth of ESCC *in vivo*. **(A)** The timeline for ESCC xenografts mouse model construction and chemotherapy strategy. **(B)** Time courses of animal body weight. **(C)** Time courses of xenograft tumor volume. **(D)** Xenograft tumor weight histogram. **(E)** Tumor inhibition rate of CEH, CDDP, and combination chemotherapy strategies. **(F)** TUNEL staining of paraffin sections of xenograft tumor tissues. Error bars represent mean ± SD. N, negative control; M, model control; CDDP, CDDP chemotherapy; CEH, cepharanthine hydrochloride chemotherapy; Comb, combined chemotherapy strategy; ns, no significant difference. ^*^P < 0.05, ^**^P < 0.01, ^***^P < 0.001.

Female BALB/c nude mice (8–10 weeks) were purchased from Beijing Vital River Laboratory Animal Technology Co., Ltd. (Certificate number: 11401300072303), and housed in a specific pathogen-free (SPF) environment with a temperature of 21 ± 2°C at a relative humidity of 30–70%, and a photoperiod of 12/12-h in the Animal Experimental Management Center of Jinan University (Experimental animal ethics certificate number: IACUC20180319-07). Eca109 cells (1 × 10^6^) with Matrigel (BD Matrigel™ 354248) were injected subcutaneously (s.c.) into the flank of the mice. When tumors grew to ~100 mm^3^ in volume, mice were randomly divided into five groups: Normal control (N), Model group (M), Cisplatin (CDDP), Cepharanthine hydrochloride (CEH), and Combination (Comb), six mice per group. The experimental procedures are shown in Figure 1A. Administration by intraperitoneal injection (i.p.): CDDP, 5 mg/kg every 5 days; CEH, 10 mg/kg daily. After the last administration, mice feces were collected, and the mice were euthanized to collect peripheral blood, small intestine, and ESCC xenograft tumors. Collected samples were quickly frozen and subsequently stored at −80°C.

### Western Blotting Assay

Proteins were extracted from ESCC xenograft tumors and small intestine tissue using Trident RIPA Lysis Buffer (GeneTex, Inc) with the addition of Halt™ Phosphatase Inhibitor Single-Use Cocktail (Thermo Scientific™) and Protease Inhibitor Cocktail (Sigma). Protein concentration was determined by BCA Protein Assay Kit (Thermo Scientific™). Antibodies for PARP (9532), Caspase-8 (9746), Caspase-9 (9502), Caspase-3 (9662), TNFR1 (3736), CYLD (8462), JNK (9252), p-JNK (4668), Apaf-1 (8969), Bax (5023), Bcl-2 (15071), Cytochrome c (4280), NF-κB (8242), and Myd88 (4283) were purchased from Cell Signaling. TLR4 antibody (ab13556) was obtained from Abcam. UltraSignal Hypersensitive ECL Western Blotting Substrate (4A Biotech, 4AW011-1000).

### Histopathology and Immunohistochemistry

The histopathological examination was performed with hematoxylin and eosin (H&E) staining. Apoptotic cells were measured by TUNEL staining. Antibodies for c-PARP (5625), Bax (5023), Bcl-2 (15071), and c-Caspase-3 (9664) were purchased from Cell Signaling, and IgA (abs120195) was obtained from Absin (China).

### 16S rDNA Amplicon Sequencing and Bioinformatics Analysis

Total genome DNA from samples was extracted using CTAB/SDS method. 16S rRNA genes of V4 region were amplified used the specific primer: 515F, 5′-GTGCCAGCMGCCGCGGTAA-3′; 806R, 5′-GGACTACHVGGGTWTCTAAT-3′. Library preparation and sequencing was carried out after PCR products mixing and purification. Sequencing libraries were generated using Ion Plus Fragment Library Kit 48 rxns (Thermo Scientific). The library quality was assessed on the Qubit@ 2.0 Fluorometer (Thermo Scientific). At last, the library was sequenced on an Ion S5TM XL platform and 400/600 bp single-end reads were generated. Quality filtering on the raw reads were performed to obtain the high-quality clean reads according to the Cutadapt (V1. 9.1, http://cutadapt.readthedocs.io/en/stable/) quality-controlled process. The reads were compared with the reference database (Silva database, https://www.arb-silva.de/) using UCHIME algorithm (UCHIME Algorithm, http://www.drive5.com/usearch/manual/uchime_algo.html) to detect chimera sequences, and then the chimera sequences were removed. Species composition was revealed by reads shear filtration, operational taxonomic units (OTUs) clustering (Uparse v7. 0.1001, http://drive5.com/uparse/, sequences with ≥97% similarity were assigned to the same OTUs), species annotation (the Silva Database, https://www.arb-silva.de/ was used based on Mothur algorithm to annotate taxonomic information), and abundance analysis. OTUs abundance information were normalized using a standard of sequence number corresponding to the sample with the least sequences. Subsequent analysis of alpha diversity and beta diversity were all performed basing on this output normalized data. Alpha diversity is applied in analyzing complexity of species diversity for a sample through 6 indices, including Observed-species, Chao1, Shannon, Simpson, ACE, Good-coverage. All these indices in our samples were calculated with QIIME (Version 1.7.0) and displayed with R software (Version 2.15.3). Two indices were selected to identify Community richness: Chao—the Chao1 estimator (http://www.mothur.org/wiki/Chao); ACE—the ACE estimator (http://www.mothur.org/wiki/Ace); Two indices were used to identify Community diversity: Shannon—the Shannon index (http://www.mothur.org/wiki/Shannon); Simpson—the Simpson index (http://www.mothur.org/wiki/Simpson); One indice to characterized Sequencing depth: Coverage—the Good's coverage (http://www.mothur.org/wiki/Coverage). Beta diversity analysis was used to evaluate differences of samples in species complexity, Beta diversity on both weighted and unweighted unifrac were calculated by QIIME software (Version 1.7.0). Cluster analysis was preceded by principal component analysis (PCA), which was applied to reduce the dimension of the original variables using the FactoMineR package and ggplot2 package in R software (Version 2.15.3). Principal Coordinate Analysis (PCoA) was performed to get principal coordinates and visualize from complex, multidimensional data. A distance matrix of weighted or unweighted unifrac among samples obtained before was transformed to a new set of orthogonal axes, by which the maximum variation factor is demonstrated by first principal coordinate, and the second maximum one by the second principal coordinate, and so on. PCoA analysis was displayed by WGCNA package, stat packages and ggplot2 package in R software (Version 2.15.3). Non-metric multidimensional scaling (NMDS) analysis was displayed by vegan package in R software. Unweighted Pair-group Method with Arithmetic Means (UPGMA) Clustering was performed as a type of hierarchical clustering method to interpret the distance matrix using average linkage and was conducted by QIIME software (Version 1.7.0). The default setting for LEfSe (LDA Effect Size) Analysis Score was 4. Data were statistically analyzed according to the specified protocols (Novogene Bioinformatics Technology Co., Ltd.).

### Metagenomic Analysis

DNA contents above 1 μg are used to construct library after passing the DNA degradation degree and potential contamination test. The DNA sample was fragmented by sonication to a size of 350 bp, then DNA fragments were end-polished, A-tailed, and ligated with the full-length adaptor for Illumina sequencing with further PCR amplification. At last, PCR products were purified (AMPure XP system) and libraries were analyzed for size distribution by Agilent2100 Bioanalyzer and quantified using real-time PCR. The clustering of the index-coded samples was performed on a cBot Cluster Generation System. After cluster generation, the library preparations were sequenced on an Illumina HiSeq platform and paired-end reads were generated. The data obtained by the Illumina Hiseq sequencing strategy were used for bioinformatics analysis, including species annotation, functional annotation, and abundance statistics based on KEGG, eggNOG, CAZy, and CARD (Novogene Bioinformatics Technology Co., Ltd.). The strategy adopted by Metagenomic Analysis is similar to that reported by Mohammad Bahram (Bahram et al., 2018).

### Myeloperoxidase (MPO) Analysis

MPO in the spleen and small intestine was detected using an MPO kit (Nanjing Jiancheng Bioengineering Institute, China), according to the manufacturer's instructions. MPO activity was expressed as units per gram of tissue (U/g).

### Real-Time Quantitative PCR Assay

The mRNA expression of TNF-α and other factors from different sources were analyzed by quantitative PCR (qPCR) using TB Green^TM^ premix Ex Taq^TM^ II (TaKaRa, RR820A) and then analyzed with a C1000 Thermal Cycler (CFX96 Real-Time System, Bio-Rad). The mRNA level of β-actin was measured as the internal control. The oligonucleotides used for qPCR in this study are displayed in Table S1.

### ELISA Analysis

Sera were collected from the peripheral blood by centrifugation at 1,000 g. The concentrations of TNF-α, TGF-β, IL-2, IL-10, IFN-β, and IL-6 in peripheral blood were measured using ELISA kits according to the manufacturers' instructions [EMC102a, EMC002, EMC005, EMC107b, EMC004, EMC016 (NeoBioscience)]. Optical densities were measured at 450 nm using a microplate reader [ELX800 (BioTek)] and a standard curve was drawn to calculate the content of immune factors.

### Statistical Analysis

Unless otherwise stated, *p*-values indicate statistical significance, calculated using the Student's *t*-test or one-way ANOVA when appropriate and denoted by ^*^ = *p* < 0.05, ^**^ = *p* < 0.01, ^***^ = *p* < 0.001. Results are representative of at least three independent experiments.

## Results

### CEH Enhances the Chemotherapy Effect of CDDP *in vivo*

In order to examine the anti-tumor effects of CEH *in vivo*, we treated the mice bearing ESCC xenograft tumors with different therapeutic strategies (Figure 1A). During treatment, we found that CEH therapy did not decrease bodyweight; however, CDDP alone or combined with CEP resulted in significant weight loss (Figure 1B). In terms of anti-tumor effects, the combination of CDDP and CEH showed a better efficacy in terms of tumor size and weight (Figures 1C,D). Compared with CDDP or CEH therapy alone, the tumor inhibition rate of combined therapy was much higher (Combined therapy, 54.75 ± 7.96%; CDDP, 29.10 ± 8.12%; CEH 25.80 ± 6.76%) (Figure 1E). H&E staining showed that cell division in the model group was vigorous, and the combined therapy resulted in greater nuclear pyknosis and cytoplasmic lysis of xenograft tumor cells compared to treatment with CEH or CDDP alone (Figure S1A). In addition, the immunohistochemical analysis showed that combined therapy induced more cellular apoptosis in xenograft tumors, as demonstrated by TUNEL staining (Figure 1F).

**Figure 2:**
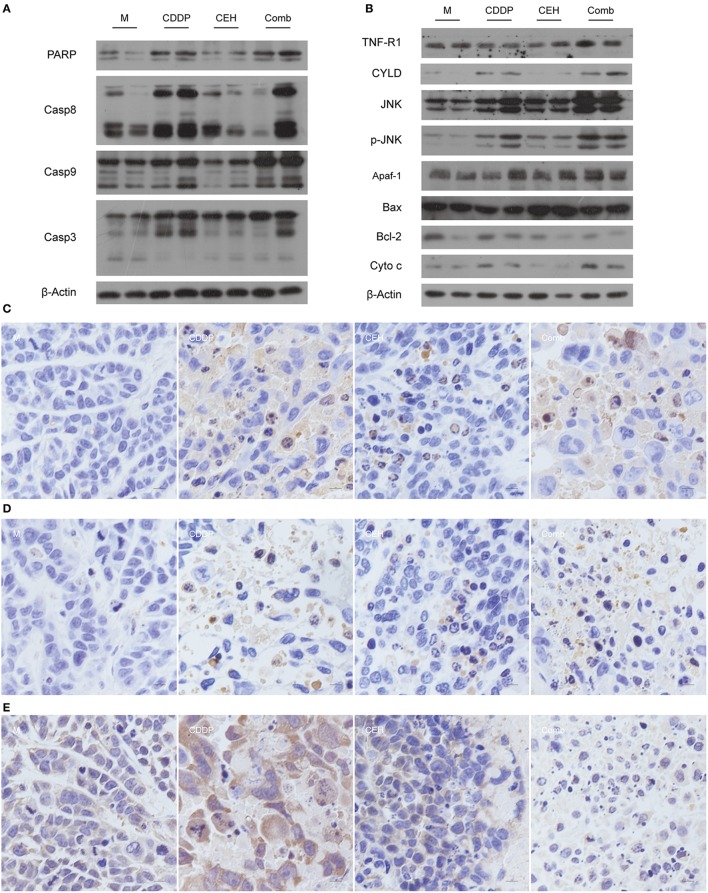
CEH combined with CDDP chemotherapy strategy can promote apoptosis. **(A)** Western blot analysis of the apoptosis-associated proteins: PARP, Casp8, Casp9, Casp3. **(B)** Western blot analysis of the apoptotic signal transduction pathway-associated proteins: TNFR, CYLD, JNK, p-JNK, Apaf-1, Bax, Bcl-2, and Cyto-c. β-Actin was used as the protein loading control. Immunohistochemical staining of c-PARP **(C)**, c-Caspase 3 **(D)**, and Bcl-2 **(E)**. N, negative control; M, model control; CDDP, CDDP chemotherapy; CEH, cepharanthine hydrochloride chemotherapy; Comb, combined chemotherapy strategy.

In order to further analyze the effects of combined therapy, we detected the apoptosis signaling pathway by western blotting. Compared with the single-use of CDDP or CEH, the combined therapy exhibited high protein levels of cleaved PARP, Caspase-8, Caspase-9, and Caspase-3 (Figure 2A), indicating that the strategy of combined therapy enhances apoptosis signaling. Mechanistically, combined therapy activated the TNFR1-JNK signal transduction axis, which induces apoptosis. Accordingly, Bcl-2 was inhibited in combined therapy. In addition, the amount of cytochrome C (Cyto C) in the combined therapy group was significantly increased (Figure 2B). Meanwhile, immunohistochemical analysis also showed that apoptosis-related proteins of c-PARP (Figure 2C), c-Caspase 3 (Figure 2D), Bcl-2 (Figure 2E), and Bax (Figure S1B) were detectable in drug-treated groups. These results suggest that combined therapy of CEH and CDDP shows better anti-tumor effects *in vivo* than CDDP alone.

### Taxonomic Shifts in the Microbes Following Different Chemotherapy Strategies

**Figure 3:**
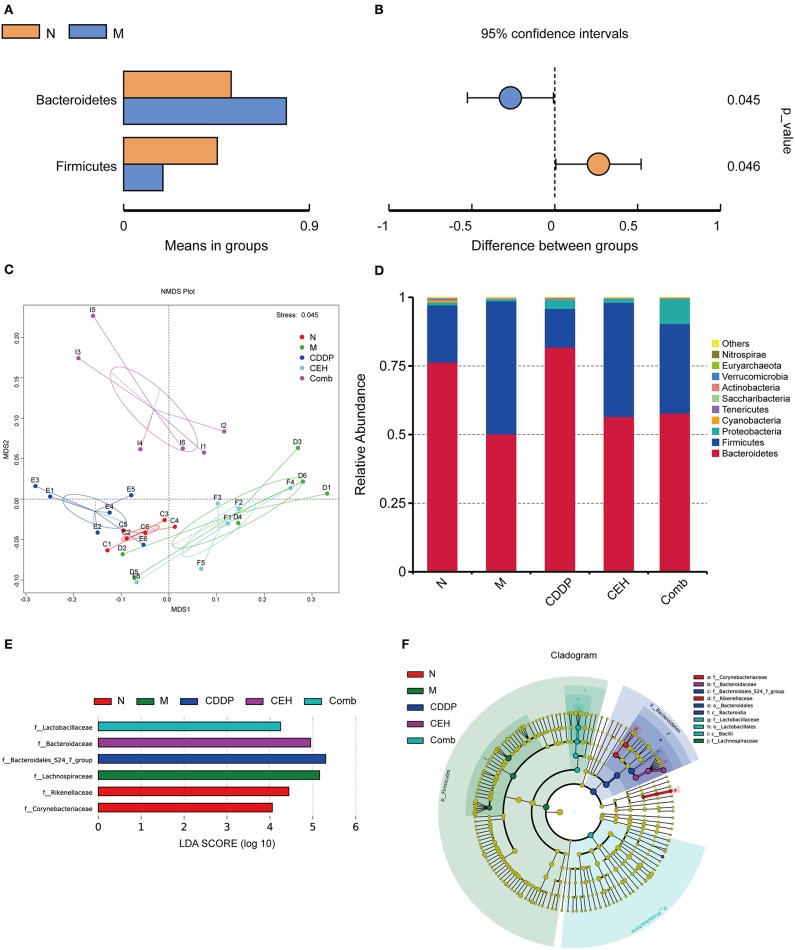
Taxonomic shifts in the microbes. *T*-test of species difference at the phylum level between N and M; **(A)** Species abundance display, mean relative abundance of Phylum which with significant difference within the group; **(B)** Difference confidence display, the difference value of the mean of relative abundance. **(C)** NMDS plots based on weighted UniFrac distance matrices depicting data based on 16S rRNA gene sequencing. **(D)** Top 10 species at the phylum level relative abundance histogram. LEfSe (LDA Effect Size) analysis of species with significant differences among different chemotherapy strategy groups. **(E)** Histogram of the LDA scores at the Family level (the default setting is 4). **(F)** Cladogram. N, negative control; M, model control; CDDP, CDDP chemotherapy; CEH, cepharanthine hydrochloride chemotherapy; Comb, combined chemotherapy strategy.

An increasing amount of evidence shows that the intestinal microbes contributes to the initiation and progression of tumors, in both intestinal and extra-intestinal distal tumors (Mima et al., 2017; Deng et al., 2018; Ma et al., 2018; Routy et al., 2018; Schramm, 2018; Tilg et al., 2018). Thus, we assessed the effect of subcutaneous xenograft tumors on the host's intestinal microbes. An average of 80,170 clean reads were obtained per sample based on 16S rDNA sequences, and then we performed an analysis of the OTUs. The xenograft tumor-bearing group showed a statistically significant increase in Bacteroidetes and a decrease in Firmicutes compared to the normal mice (Figures 3A,B). NMDS plots of these data are shown in Figure 3C. Simultaneously, we also found that different chemotherapy strategies can affect the intestinal microecological structure in mice. The top 10 communities were Bacteroidetes, Firmicutes, Proteobacteria, Cyanobacteria, Tenericutes, Saccharubacteria, Actinobacteria, Verrucomicrobia, Euryarchaeota, and Nitrospirae at the phylum level (Figure 3D). As shown in Figures 3E,F, we used linear discriminant analysis (LDA) of effect size (LEfSe) to determine the representative differences in taxa after different chemotherapy strategies. Furthermore, we obtained genus level abundance cluster heatmaps and relative abundance histograms of the top 10 species, as shown in Figures S2A,B, respectively. Corynebacterium and Alistipes were enriched in the normal control group; however, Lachnospiraceae_NK4A163_group and unidentified_Lachnospiraceae were enriched in the tumor-bearing model group at the genus level. After chemotherapy, Bacteroidales_S24_7_group was found to be enriched in the CDDP group, Bacteroidaceae was enriched in the CEH group, and Lactoacillaceae in the Comb group, at the family level. It is worth noting that two intestinal bacteria, Bacterides_acidifaciens and Acinetobacter_johnsonii, were enriched in the CEH treatment alone group, at the species level. These results highlight the intestinal microecological changes under different chemotherapy strategies.

### CEH Rescues Intestinal Microbes Disturbance Caused by CDDP

**Figure 4:**
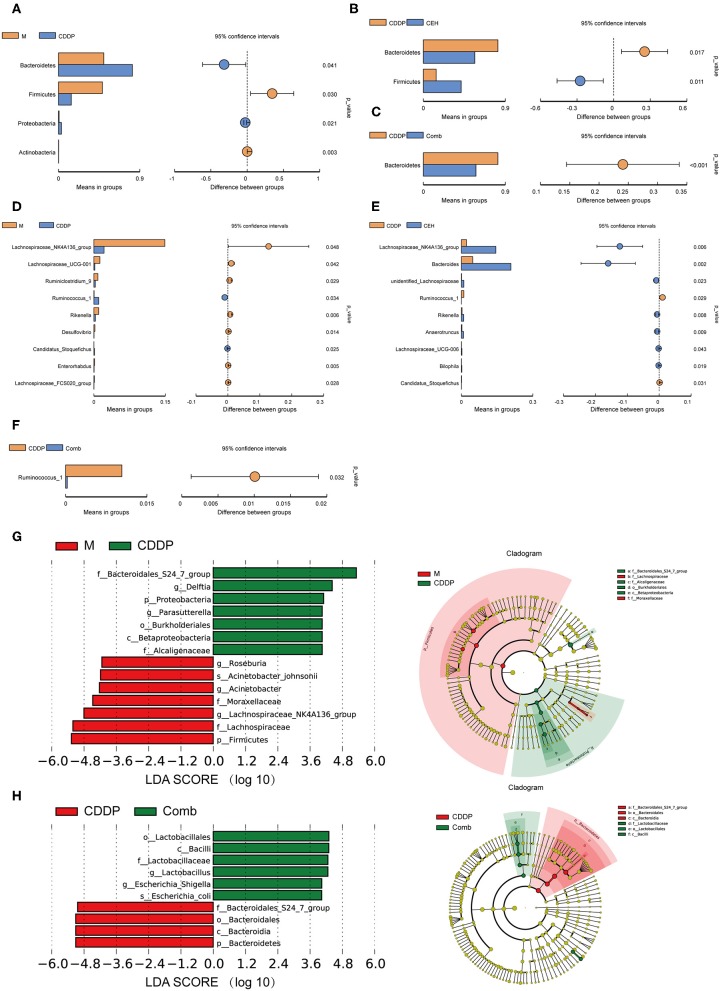
Combination of CEH with CDDP can improve the intestinal microbes structural changes caused by chemotherapy. **(A–C)** *T*-test of species differences at the phylum level. **(A)** Comparison between M and CDDP. **(B)** Comparison between CDDP and CEH. **(C)** Comparison between CDDP and Comb. **(D–F)** *T*-test of species differences at the genus level. **(D)** Comparison between M and CDDP. **(E)** Comparison between CDDP and CEH. Mean relative abundance of species which with significant difference within the group (**A–E** left panel). Difference confidence display, the difference value of the mean of relative abundance (**A–E** right panel). **(F)** Comparison between CDDP and Comb. **(G)** LEfSe (LDA Effect Size) analysis of species with significant differences between M and CDDP. **(H)** LEfSe analysis of species with significant differences between CDDP and Comb. N, negative control; M, model control; CDDP, CDDP chemotherapy; CEH, cepharanthine hydrochloride chemotherapy; Comb, combined chemotherapy strategy.

In order to further evaluate the effects of different chemotherapy strategies on intestinal microbes in tumor-bearing mice at different classification levels (phylum, genus, and species), the amount of species was determined and analyzed statistically. CEH increased the amount of Parasutterella and Parabacteroides (Figure S2H) relative to the model control group. Additionally, the CEH and CDDP combined chemotherapy strategy reduced the amount of Bacteroides, Alistipes, and Rikenella (Figures S2F,G) when compared with the normal and model controls. These results provide strong evidence that CEH regulates the intestinal microbes living in model and CDDP-treated mice. Furthermore, we investigated different effects of CEH and CDDP on intestinal microbes abundance. Compared with the normal control group, the amount of Lactobacillus in the model group was significantly reduced and was further reduced in the CDDP group (Figures S2C,D). Conversely, CEH significantly increased the amount of Bacteroides, which play a fundamental role in the processing of complex molecules to simpler ones in the host intestine (Wexler, 2007) (Figure S2E). On the contrary, CDDP therapy alone increased the amount of Bacteroidetes and decreased the amount of Firmicutes compared to the model controls, at the phylum level (Figure 4A). Interestingly, CEH induced a reversed distribution pattern from the above, as shown in Figures 4B,C. At the genus level, CDDP chemotherapy decreased the amount of Lachnospiraceae_NK4A163_group and Lachnospiraceae_UCG-001 (Figure 4D), while CEH further increased the amount of Lachnospiraceae_NK4A163_group (Figure 4E) but reduced the amount of Ruminococcus_1 (Figure 4F). These results represented some intestinal microbes shifts as a result of CEH and CDDP therapies. At the same time, Lachnospiraceae_NK4A163_group, Bacteroides, Alietipes, and Rikenella (Figure S2I) were enriched in the CEH and the combination chemotherapy strategy groups, however, they showed higher amounts in the CEH group. We then investigated the key phylotypes between groups using LEfSe analysis. Bacteroidales_S24_7_group, Lachnospiraceae_UCG_010, and Bacteroidaceae were more abundant in the normal control group, the tumor-bearing model group, and the CDDP chemotherapy group, respectively (Figures S2J,K). Homoplastically, Lachnospiraceae, Moraxellaceae, Alcaligenaceae, and Bacteroidales_S24_7_group were identified as the main contributors to the differences in the fecal microbes' structure between the tumor bearing model group and the CDDP chemotherapy group (Figure 4G). However, the Bacteroidales and Lactobacillaceae microbes communities were found to be significantly different between the CDDP chemotherapy group and the combination chemotherapy strategy group (Figure 4H). Moreover, we also isolated several unique species, including Bacteroides_acidifaciens, Acinetobacter_johnsonii, Escherichia_shigella, and Lachnospiraceae_bacterium_615, in the CEH chemotherapy group (Figure S2L). Collectively, these results demonstrate that different therapies result in changes in the intestinal microbes of mice. CEH has the capacity to increase the proportions of beneficial bacteria, such as Lactobacillus, and exerts a regulatory function on intestinal microbes.

### Different Chemotherapy Strategies Change the Function of Intestinal Microbes

**Figure 5:**
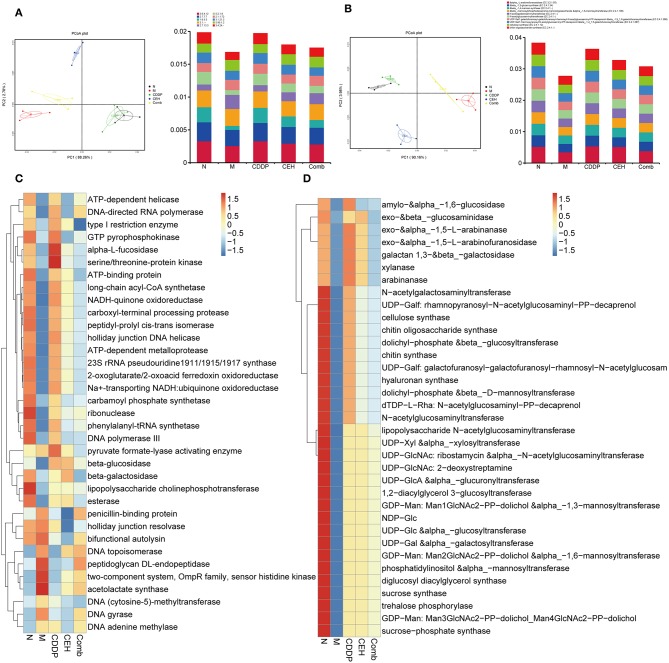
Different chemotherapy strategies caused changes in the function of the intestinal microbes. **(A)** PCoA analysis based on KEGG EC (enzyme) abundance (left panel) and top 10 abundance histogram (right panel). **(B)** PCoA analysis based on CAZy EC abundance (left panel) and top 10 abundance histogram (right panel). **(C)** KEGG EC top 35 abundance clustering heat map. **(D)** CAZy EC top 35 abundance clustering heat map. N, negative control; M, model control; CDDP, CDDP chemotherapy; CEH, cepharanthine hydrochloride chemotherapy; Comb, combined chemotherapy strategy.

After determining the effect of each chemotherapy strategy on the structure of the intestinal microbes, we performed metagenomic analyses using the IlluminaHiseqPE sequencing platform to identify the changes in microbes function during anticancer treatment. Based on the Kyoto Encyclopedia of Genes and Genomes (KEGG) and the Carbohydrate-Active enZYmes (CAZy) database, we analyzed changes in the bio-metabolic pathway and carbohydrate metabolism of intestinal microbes after different therapy strategies. First, PCoA dimensionality reduction analysis based on KO and CAZy abundance was used to characterize the differences in the enzymes (Figures 5A,B) and function (Figures S3A,B) between the different treatment groups. In order to better understand the functional changes in the different chemotherapy strategy groups, a differential function clustering heat map was drawn by selecting the top 35 KO metabolic pathways (Figure S3C) and CAZy's second-level sub-functions (Figure S3D) based on the functional annotations and abundance information of all samples. Similarly, we clustered the top 35 enzymes in the KEGG metabolic pathways (Figure 5C) and CAZy (Figure 5D). The results show that different therapy strategies have a significant impact on multiple biological processes, including cell motility, replication and repair, energy metabolism, membrane transport, carbohydrate metabolism, nucleotide metabolism, translation, glycan biosynthesis and metabolism, amino acid metabolism, folding, sorting and degradation, metabolism of cofactors and vitamins, signal transduction, and cellular community in prokaryotes. Compared with the xenograft control group, CEH improved the dysfunction caused by CDDP therapy in terms of the starch and sucrose metabolism, glycan biosynthesis and metabolism, the energy metabolism, the amino sugar and nucleotide sugar metabolism, cellular community-prokaryotes-Quorum sensing, bacterial chemotaxis, flagellar assembly, phenylalanine, tyrosine, and tryptophan biosynthesis, and the bacterial secretion system. At the same time, we found that the effects of different chemotherapy strategies on carbohydrate metabolizing enzymes are also significant. Compared with control group, important changes occurred in the xenotransplantation model group, both in function of the intestinal microbes and in carbohydrate enzymes.

### CEH Improves Intestinal Mucosal Immunity

**Figure 6:**
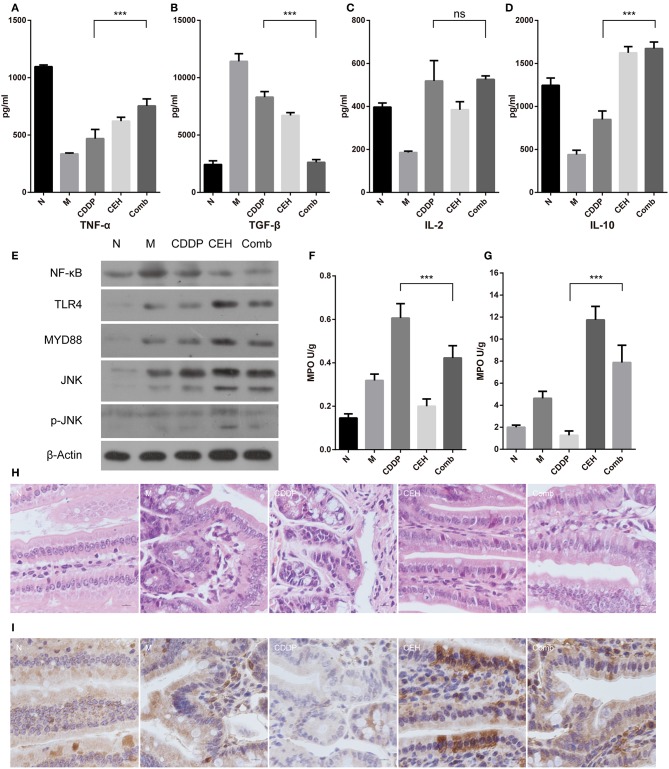
CEH enhances intestinal mucosal immunity. ELISA detected the peripheral blood immune factors, TNF-α **(A)**, TGF-β **(B)**, IL-2 **(C)**, and IL-10 **(D)**. **(E)** Western blot analysis of the TLR4 signaling pathway-associated proteins in small intestine tissue: NF-κB, TLR4, Myd88, JNK, and p-JNK. β-Actin was used as the protein loading control. MPO analysis was used to characterize neutrophil aggregation in the small intestine **(F)** and spleen **(G)**. **(H)** HandE staining of paraffin sections for small intestine tissue. **(I)** Immunohistochemical staining of IgA. Error bars represent mean ± SD. N, negative control; M, model control; CDDP, CDDP chemotherapy; CEH, cepharanthine hydrochloride chemotherapy; Comb, combined chemotherapy strategy; ns, no significant difference. ^***^P < 0.001.

The complex composition of the gut microbiota is now recognized to be strongly involved in host metabolic and immunologic homeostasis. Since immune homeostasis is crucial in tumor chemotherapy, we used ELISA to detect the immune factors in the peripheral blood. We found that the combined therapy increased TNF-α and IL-10 levels (Figures 6A,D), while reducing TGF-β levels (Figure 6B). IL-2 levels did not change (Figure 6C). Furthermore, the combined therapy increased the expression of TNF-α, TNFR1, IFN-α/β, Ifngr1, and OSM in the model group and reduced the expression of EGF, EGFR, and TGF-β (Figure S4). Mechanically, CEH activated TLR4 and MYD88 innate immune signaling, which is advantageous for the activation of the host's innate immunity, for a balanced intestinal environment as well as to exert a chemotherapeutic response to esophageal cancer. In addition, TNFR death receptors were activated by their respective ligands to induce apoptosis. Interestingly, we found that NF-κB was down-regulated in the combined therapy group (Figure 6E). Neutropenia is a major toxic side effect of chemotherapeutics, which may easily lead to intestinal microbes translocation and secondary infection. Myeloperoxidase (MPO), a functional and activating marker of neutrophils, was reduced in the small intestine and increased in spleen of the combined therapy group, reversing the side effects of CDDP in these two organs (Figures 6F,G). Intestinal mucosal immunity, as part of the intestinal barrier, plays an important role in defending against pathogenic bacteria and in establishing the relative stability of intestinal microecology. Therefore, we examined the general intestinal morphology by H&E staining. CDDP loosed the tight junctions of the cells, destroyed intestinal mucosal integrity, and shallowed the crypts in the small intestine (Figure 6H). In the combined therapy group, all of the above changes in the CDDP group were effectively improved. Secretory IgA is important for maintaining the mucosal barrier and protecting against pathogens. Immunohistochemistry staining for IgA showed that CEH significantly increased IgA (Figure 6I and Figure S4C), where even the down-regulated IgA caused by CDDP was improved by combined therapy. These results highlight the beneficial role of CEH in host immunity during chemotherapy with CDDP, which may also contribute to its anti-tumor effects.

## Discussion

An imbalance in the intestinal microecology is known to cause adverse reactions. Hence, we aimed to identify an effective strategy to alleviate the intestinal microenvironment imbalance caused by chemotherapy. A growing body of evidence is showing that the gut microbiota modulates the host response to chemotherapeutic drugs, with three main clinical outcomes: facilitation of drug efficacy; abrogation and compromise of anticancer effects; and mediation of toxicity (Alexander et al., 2017). Many anticancer drugs possess antibiotic activities, which play a part in cytotoxic activities and cause extensive damage to the intestinal mucosa (Perales-Puchalt et al., 2018). However, the effects of CDDP on intestinal mucosal immunity and intestinal microbes' structure have rarely been reported. Some medicinally important insects, medicinal plants, medicinal mineral-derived compounds, and derivatives exhibit effectiveness, low costs, safety, and regulatory effects on the intestinal microbes (Wang et al., 2012; Habtemariam, 2016; Sreedhar et al., 2016). Our previous study revealed that CEH, which is a plant-derived compound derivative, can act as an agent for reversing the resistance of CDDP (Zhou et al., 2017), while other studies indicate an immunoregulatory role (Uto et al., 2011). Therefore, in this study, we investigated the effect of CDDP chemotherapy on intestinal microbes and the intestinal mucosal immune system in mice with ESCC, as well as the protective role of CEH during CDDP chemotherapy. To the best of our knowledge, this is the first study to evaluate the role of CEH in regulating intestinal mucosal immunity as well as intestinal microbes' structure and function, during CDDP chemotherapy. This research may provide a new scientific basis for the chemotherapy strategy of CDDP combined CEH.

The main focus of oncomicrobiome research to date has been on the microbes' role in the etiology of cancer and cancer risk (Holmes et al., 2012). Evidence indicates that microorganisms in the gastric mucosa of a normal esophagus, squamous cell carcinoma, and reflux esophagitis are different. At the same time, the gastrointestinal microbes have been found to play an important role in the initiation and progression of ESCC (Nasrollahzadeh et al., 2015). Similar results were found by our research, where in normal control mice and tumor model mice, that is in the tumor-bearing model group, Bacteroidetes increased and Firmicutes decreased. Interestingly, in CEH-treated mice, Lachnospiraceae_NK4A163_group was dominant at the genus level, which may produce histone deacetylation inhibitor butyric acid to protect the body from tumorigenesis (Ruan, 2013; Meehan and Beiko, 2014; van de Wouw et al., 2018). Increasing evidence from human, animal, and *in vitro* studies suggest that gut bacteria are closely linked to the pharmacological effects of chemotherapies (CDDP, 5-fluorouracil) and immunotherapies (anti-PD-L1, anti-CLTA-4) (Alexander et al., 2017; Perales-Puchalt et al., 2018). Consistent with previous reports (Montassier et al., 2015), our findings also showed maladjusted Bacteroidetes to Firmicutes ratios and loss of Lachnospiraceae_NK4A163_group caused by CDDP therapy. Furthermore, CDDP exhibits a selective antibiotic effect that reduces specific bacterial species (e.g., Lachnospiraceae), allowing for an increase in the relative abundance of others (e.g., Bacteroidales). More importantly, CEH can partially restore these changes.

Disorder of the intestinal microbes community can affect the carbohydrate metabolism, as well as amino acids and nucleotides metabolism, and thus change the host's immune response homeostasis (Sun et al., 2015; Endesfelder et al., 2016; Mathewson et al., 2016; Zhernakova et al., 2018). In terms of metabolism, by metagenomic analysis, we found that CDDP chemotherapy significantly affects the complex carbohydrate metabolism and the associated signaling pathways. For example, CDDP activates the alpha-L-fucosidase pathway (3.2.1.51) (Table S2), which is commonly used as an indicator for early diagnosis of primary liver cancer. However, the two-component system (2.7.13.3) (Table S2), bacterial chemotaxis, ABC transporters, quorum sensing, and starch and sucrose metabolisms (Table S4) are inhibited by CDDP chemotherapy. Chemotherapy with CDDP alters the original state of the glucose metabolism, wherein it decreases mannosyl-glycoprotein endo-β-N-acetylglucosaminidase and increases mannosyl-oligosaccharideα-1,2-mannosidase (Tables S3, S5), which were previously associated with a chemotherapy-induced inflammatory environment (Ramos et al., 2004). These discoveries may be useful for the development of new strategies to manage gastrointestinal mucositis due to chemotherapy.

Carbohydrate fermentation is a core activity of the human gut microbiota, driving the energy and carbon economy of the colon. The dominant and prevalent species of gut bacteria, including SCFA-producers, appear to play a critical role in the initial degradation of complex plant-derived polysaccharides (El Kaoutari et al., 2013). The conservation of the gut microbes may therefore improve not only tumor- and treatment-associated changes in the gut but also systemic metabolic disturbances. CEH was found to significantly raise the relative abundance of Bacteroides, which able to ferment glucose, lactose, maltose, fructose, raffinose, arabinose, cellobiose, rhamnose, and other carbohydrates. This is important for both metabolism and host immune regulation (Chan et al., 2018). The complex composition of the gut microbiota is now recognized to be strongly involved in host metabolic and immunologic homeostasis. The local immune system faces the daunting task of enforcing a harmonious coexistence with these microbes while also imposing a staunch barrier to prevent pathogen invasion (Perez-Lopez et al., 2016). Abuse of antibiotics, chemotherapy, and long-term chronic inflammatory responses destroy the steady-state relationship between the gut microbiota and the intestinal mucosal immune system, leading to symptoms of loss of appetite, nausea, vomiting, abdominal pain, chemotherapy-induced diarrhea, and infection (Wei et al., 2018). In this process, intestinal mucosal cell damage and intestinal microbes' imbalance synergistically lead to intestinal mucosal immune dysfunction. Immunoglobulin A (IgA), the main antibody secreted by the intestinal mucosa, is an important contributor to intestinal barrier function and modulates the composition of the microbiota (Johansen et al., 1999; Suzuki et al., 2004; Peterson et al., 2007). CEH combined with the CDDP chemotherapy strategy can improve the loss of IgA during CDDP chemotherapy alone (Figure 6I). During this process, the innate immune effector molecules tumor necrosis factor α (TNFα) is also upregulated. These changes are protective responses exhibited by some B lineage cells and are important for maintaining intestinal homeostasis (Fritz et al., 2011). On the contrary, the intestinal microbes may modulate the activation of NF-κB, potentially favoring inflammation. Our results showed that CEH down-regulated the expression of NF-κB, which may be related to its own anti-inflammatory effect. Meanwhile, CEH up-regulated the relative abundance of some intestinal bacteria, such as Lachnospira, which attenuated inflammation by modulation of the NF-κB pathway (Lakhdari et al., 2011; Montassier et al., 2015). This also reflects the fact that CEH can improve the intestinal mucosal immune disorder caused by chemotherapy.

The response to innate immunity allows the body to fight infection and maintain a balanced microenvironment in the gut, a process which plays an important role in mucosal immune homeostasis, thus protecting against intestinal epithelial damage, regulating intestinal motility, and distinguishing between pathogens and commensal microorganisms (Rakoff-Nahoum et al., 2004; Anitha et al., 2012; van Egmond et al., 2015; Jiménez-Dalmaroni et al., 2016; Satoh and Akira, 2016). TLRs are pattern-recognition receptors that play a crucial role in initiating the innate immune response against microbes for fighting infection and maintaining homeostasis, both (Liu et al., 2014). The commensal microbes is critical in maintaining intestinal mucosal immunity and TLR4 expression in the intestinal mucosa (Wu et al., 2014). After recognition of PAMPs, such as polysaccharides produced by Bacteroides which were enriched in the CEH group (Figure 4E) (Sjögren et al., 2009; Jiang et al., 2017), the downstream adaptor molecule Myd88 can mediate the activation of intracellular signal transduction to trigger an immune response (Figure 6E) (Yamamoto et al., 2003; Kawai and Akira, 2010). Moreover, the activation of TLR4 enhances IgA production (Shibata et al., 2018), which is important for host resistance to chemotherapy-induced gastrointestinal mucositis.

Overall, we found that CEH combined with CDDP regulating the intestinal microbes to activate intestinal mucosal immunity and enhance the effect of chemotherapy in mice. CEH can regulate intestinal microenvironmental disorders caused by chemotherapy by adjusting the structure and function of intestinal microbes, activating TLR4-MYD88 innate immune signaling, activating the death receptor signaling interactive pathway, altering the esophageal cancer chemotherapeutic response, as well activating death receptors TNFR by the respective ligands to induce apoptosis.

The gut microbiota undoubtedly plays a critical role in the development of precision treatment strategies for cancer and are increasingly being considered as a target for next-generation cancer therapies. In the future, the gut microbiota is most likely to become a central element of personalized cancer treatment strategies. In conclusion, our findings indicate that the strategy of CEH combined with CDDP chemotherapy could provide a more favorable approach to maintain intestinal microenvironment homeostasis than does CDDP chemotherapy alone.

## Ethics Statement

Laboratory Animal Ethics Committee Jinan University.

## Author Contributions

PZ carried out most of the studies and performed statistical analysis, as well as designing the study and writing the manuscript. ZL carried out the collection and/or assembly of references, their interpretation, participated in manuscript writing, and provided technical assistance with several protocols. DX and YinW performed data analysis and interpretation. QB, YF, and GS participated in the animal experiments. PC, YaW, and HL analyzed the results and revised the manuscript and results. XW, RZ, and YifW performed the conception, design, interpretation, and final approval of the manuscript. PZ, ZL, and DX contributed equally to this article. All authors read and approved the final manuscript.

### Conflict of Interest Statement

The authors declare that the research was conducted in the absence of any commercial or financial relationships that could be construed as a potential conflict of interest.

## Supplementary Material

Figure S1 **(A)** HandE staining of paraffin sections for xenograft tumor tissues. **(B)** Immunohistochemical staining of Bax.

Figure S2 **(A)** Abundance cluster heatmap at the genus level. **(B)** Top 10 species relative abundance histogram. **(C–I)** *T*-test of species difference at the genus level. **(C)** Comparison between N and M. **(D)** Comparison between N and CDDP. **(E)** Comparison between N and CEH. **(F)** Comparison between N and Comb. **(G)** Comparison between M and Comb. **(H)** Comparison between M and CEH. **(I)** Comparison between CEH and Comb. **(J)** LEfSe (LDA Effect Size) analysis of species with significant differences between N and CDDP. **(K)** LEfSe analysis of species with significant differences between N and M. **(L)** LEfSe analysis of species with significant differences between CDDP and CEH.

Figure S3 **(A)** PCoA analysis based on KO EC (enzyme), abundance (left panel), and top 10 abundance histogram (right panel). **(B)** PCoA analysis based on CAZy level 1 abundance (left panel) and top 10 abundance histogram (right panel). **(C)** KEGG level 3 top 35 abundance clustering heat map. **(D)** CAZy level 2 top 35 abundance clustering heat map. N, negative control; M, model control; CDDP, CDDP chemotherapy; CEH, cepharanthine hydrochloride chemotherapy; Comb, combined chemotherapy strategy.

Figure S4 Real-time quantitative PCR assay of TLR4 **(A)**, Myd88 **(B)**, IgA **(C)** in small intestinal tissue, and TGF-β **(D)**, TNF-α **(E)**, TNFR1 **(F)**, EGF **(G)**, EGFR **(H)**, IFN-α **(I)**, IFN-β **(J)**, Ifngr1 **(K)**, OSM **(L)** in tumor. The mRNA level of β-actin was measured as the internal control. Error bars represent mean ± SD. ^**^*P* < 0.01; ^***^*P* < 0.001.

Table S1 The oligonucleotides used for qPCR in this study.

Table S2 KEGG EC (enzyme) information (top 35).

Table S3 CAZy EC (enzyme) information (top 35).

Table S4 KEGG level 3 abundance information (top 35).

Table S5 CAZy level 2 abundance information (top 35).
