# Supplementary figures and images for: Cepharanthine Hydrochloride Improves Cisplatin Chemotherapy and Enhances Immunity by Regulating Intestinal Microbes in Mice
Source: Front Cell Infect Microbiol. 2019 Jun 26;9:225. doi: 10.3389/fcimb.2019.00225 (PMC6606789; doi:10.3389/fcimb.2019.00225)

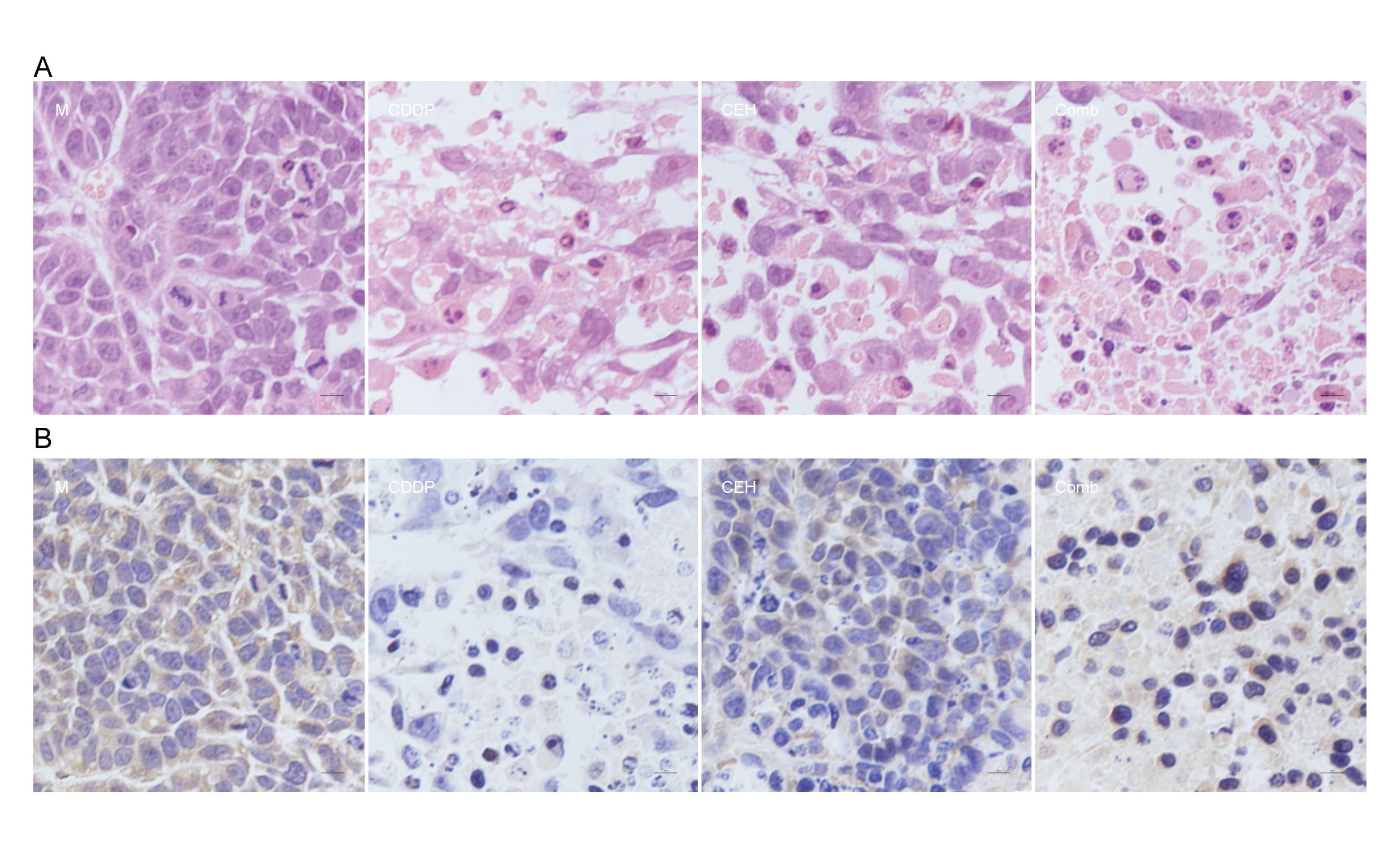

Supplement: Figure S1 — (A) HandE staining of paraffin sections for xenograft tumor tissues. (B) Immunohistochemical staining of Bax. [file Image_1.TIF]

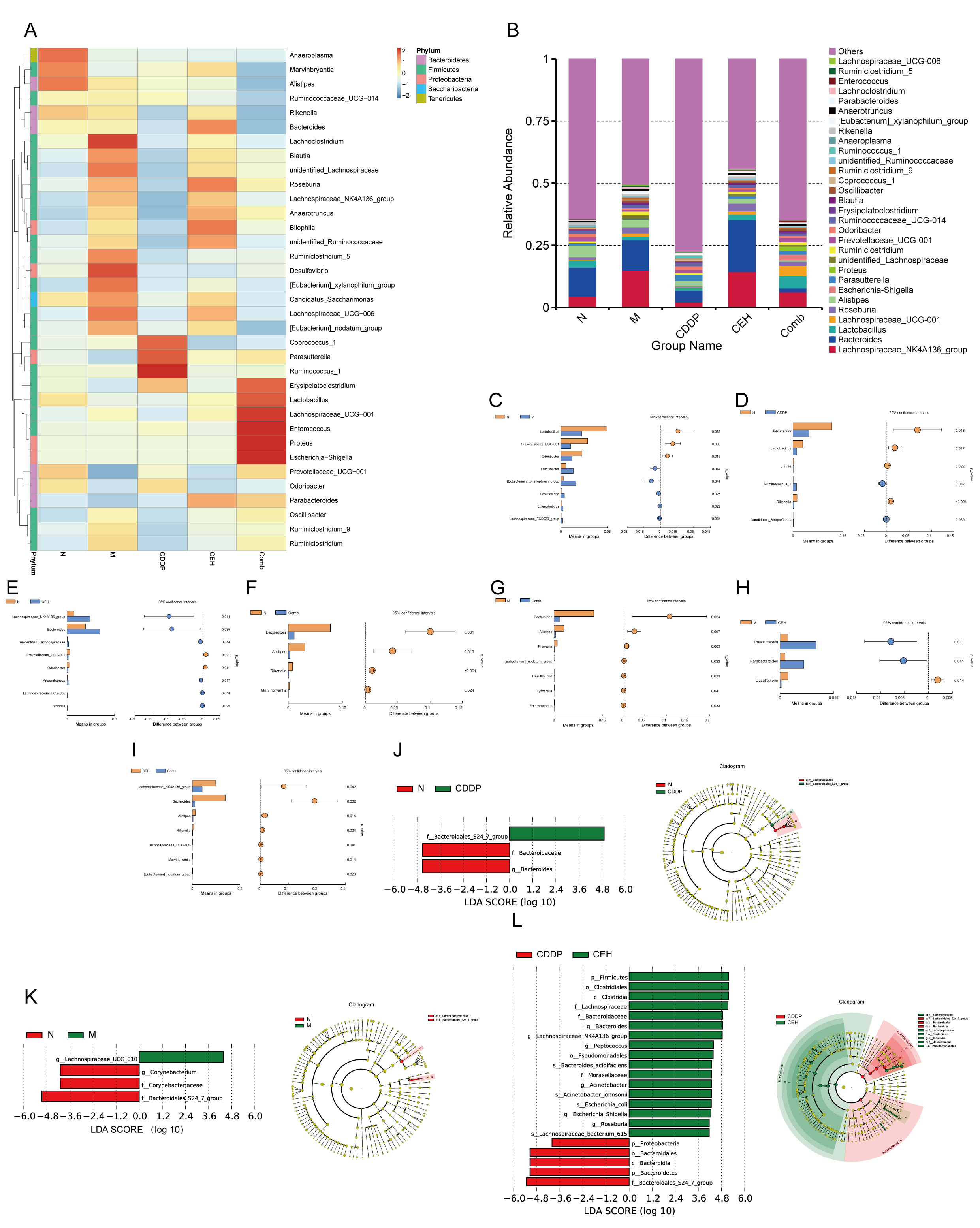

Supplement: Figure S2 — (A) Abundance cluster heatmap at the genus level. (B) Top 10 species relative abundance histogram. (C–I) T-test of species difference at the genus level. (C) Comparison between N and M. (D) Comparison between N and CDDP. (E) Comparison between N and CEH. (F) Comparison between N and Comb. (G) Comparison between M and Comb. (H) Comparison between M and CEH. (I) Comparison between CEH and Comb. (J) LEfSe (LDA Effect Size) analysis of species with significant differences between N and CDDP. (K) LEfSe analysis of species with significant differences between N and M. (L) LEfSe analysis of species with significant differences between CDDP and CEH. [file Image_2.TIF]

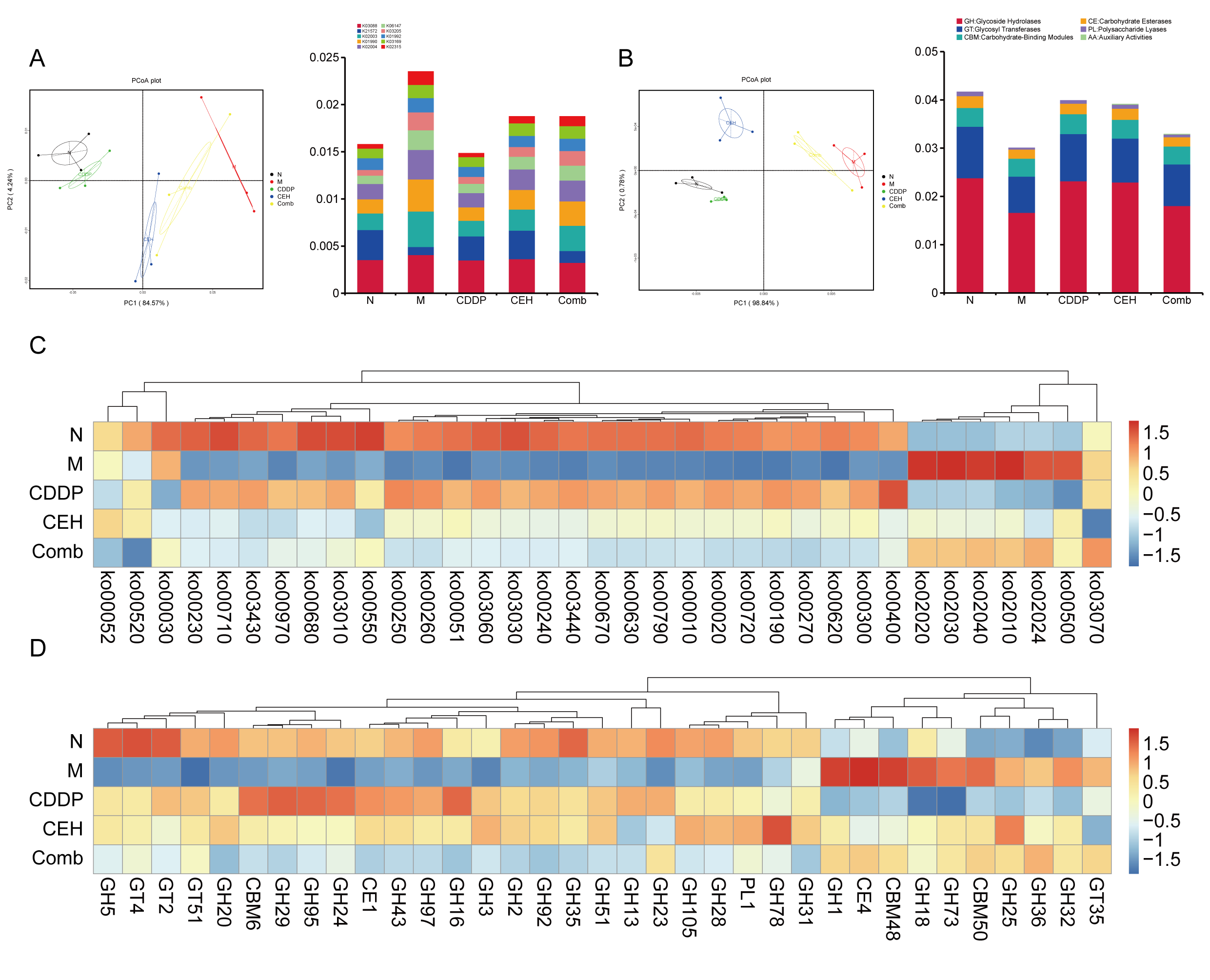

Supplement: Figure S3 — (A) PCoA analysis based on KO EC (enzyme), abundance (left panel), and top 10 abundance histogram (right panel). (B) PCoA analysis based on CAZy level 1 abundance (left panel) and top 10 abundance histogram (right panel). (C) KEGG level 3 top 35 abundance clustering heat map. (D) CAZy level 2 top 35 abundance clustering heat map. N, negative control; M, model control; CDDP, CDDP chemotherapy; CEH, cepharanthine hydrochloride chemotherapy; Comb, combined chemotherapy strategy. [file Image_3.TIF]

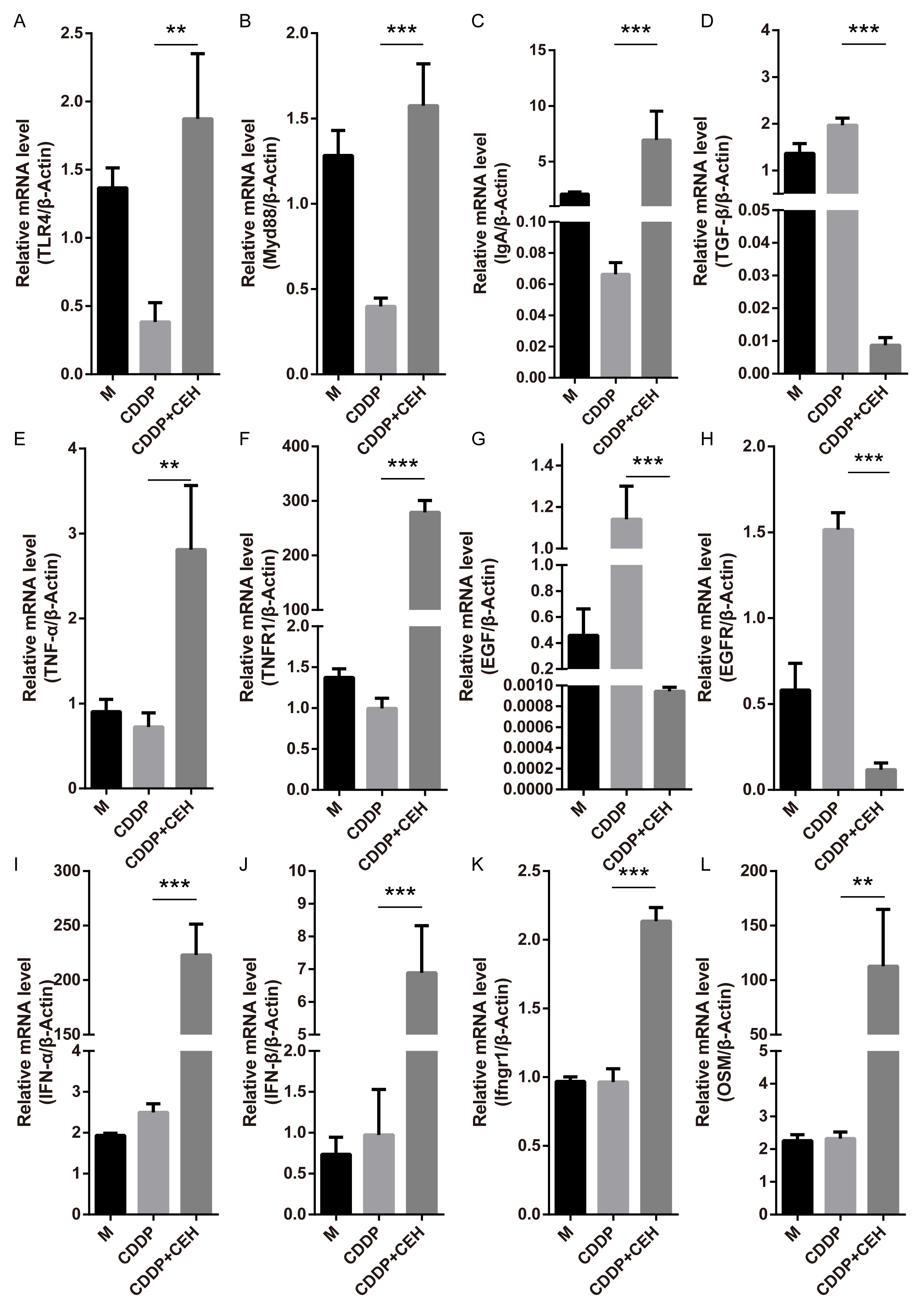

Supplement: Figure S4 — Real-time quantitative PCR assay of TLR4 (A), Myd88 (B), IgA (C) in small intestinal tissue, and TGF-β (D), TNF-α (E), TNFR1 (F), EGF (G), EGFR (H), IFN-α (I), IFN-β (J), Ifngr1 (K), OSM (L) in tumor. The mRNA level of β-actin was measured as the internal control. Error bars represent mean ± SD. **P < 0.01; ***P < 0.001. [file Image_4.tif]
